# Impact of Hypoxia-Inducible Factor Prolyl Hydroxylase Inhibitor on Renal Function in Patient with Heart Failure

**DOI:** 10.3390/jcdd8120189

**Published:** 2021-12-17

**Authors:** Teruhiko Imamura, Yohei Ueno, Koichiro Kinugawa

**Affiliations:** The Second Department of Internal Medicine, University of Toyama, 2630 Sugitani Toyama, Toyama 930-0194, Japan; fef6ge@gmail.com (Y.U.); kinugawa-tky@umin.ac.jp (K.K.)

**Keywords:** heart failure, chronic kidney disease, kidney

## Abstract

Hypoxia-inducible factor prolyl hydroxylase (HIF-PH) inhibitor is a recently introduced oral agent to treat renal anemia, but its clinical implications on renal functioning in patients with heart failure remains unknown. We studied an 81-year-old man with heart failure with mildly reduced ejection fraction, chronic kidney disease, and renal anemia. The seven-month HIF-PH inhibitor daprodustat treatment improved the hemoglobin level from 7.4 g/dL to 11.8 g/dL and estimated glomerular filtration ratio from 24 mL/min/1.73 m^2^ to 35 mL/min/1.73 m^2^ without any complications, including thromboembolic events. HIF-PH inhibitor might be a promising therapeutic tool to improve renal anemia and renal function in patients with heart failure, although large-scale studies are warranted to validate our findings.

## 1. Introduction

A considerable association among anemia, chronic kidney disease, and heart failure is known as “cardio-renal-anemia syndrome”, in which the existence of one disease worsens the others and forms a vicious cycle among them [1]. The progression of chronic kidney disease decreases hemoglobin level due to a reduction of erythropoietin secretion, which is called renal anemia [2]. The existence of anemia, on the contrary, deteriorates renal function and is an independent risk factor of mortality in patients with chronic kidney disease [3].

Recently, hypoxia-inducible factor prolyl hydroxylase (HIF-PH) inhibitor, which is orally administered and increases erythropoietin and other related factors by stabilizing HIF-alpha, has been introduced to treat renal anemia [4]. A phase III trial demonstrated a non-inferiority of HIF-PH inhibitor to conventional erythropoietin stimulating agents in improving renal anemia [5]. 

However, the clinical impact of HIF-PH inhibitor in improving renal function in patients with heart failure and chronic kidney disease remains unknown.

## 2. Case Report

### 2.1. On Admission

An 81-year-old man was referred and admitted to our institute complaining of bilateral leg pitting edema and dyspnea on efforts for several weeks. He did not have any previous history of hospitalization. He had received azosemide 60 mg/day. The New York Heart Association functional class was III.

**Figure 1:**
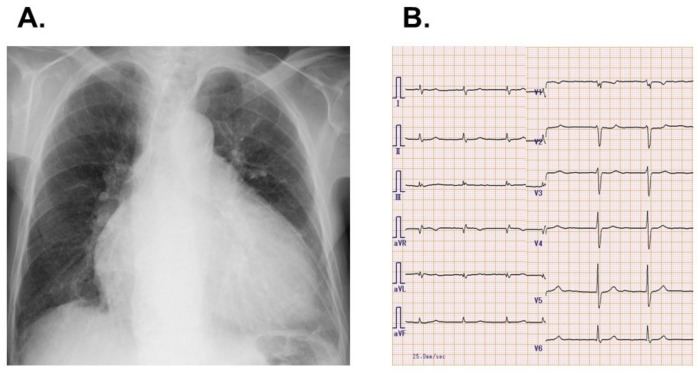
Chest X-ray (**A**) and electrocardiogram (**B**) on admission.

Body height was 153 cm and body weight was 52 kg. Blood pressure was 146/81 mmHg and pulse rate was 57 bpm. Saturation was 99% at room air. Chest X-ray showed 62% of cardiothoracic ratio and mild pulmonary congestion (Figure 1A). An electrocardiogram showed heart rate 76 bpm with atrial fibrillation and no ST-segment change (Figure 1B). Transthoracic echocardiography showed left ventricular end-diastolic diameter 56 mm and left ventricular ejection fraction 46%. Left atrial diameter was 57 mm. The degree of mitral regurgitation and tricuspid regurgitation was moderate. The diameter of inferior vena cava was 25/22 mm with a slight respiratory change.

Estimated glomerular filtration ratio was 24 mL/min/1.73 m^2^ with no proteinuria. Plasma B-type natriuretic peptide was 578 pg/mL. Hemoglobin was 7.4 g/dL. Erythropoietin was 65.2 mIU/mL, ferritin was 35 ng/mL, transferrin saturation was 10.4%, and reticulocyte was 0.4%.

### 2.2. In-Hospital Course

**Figure 2:**
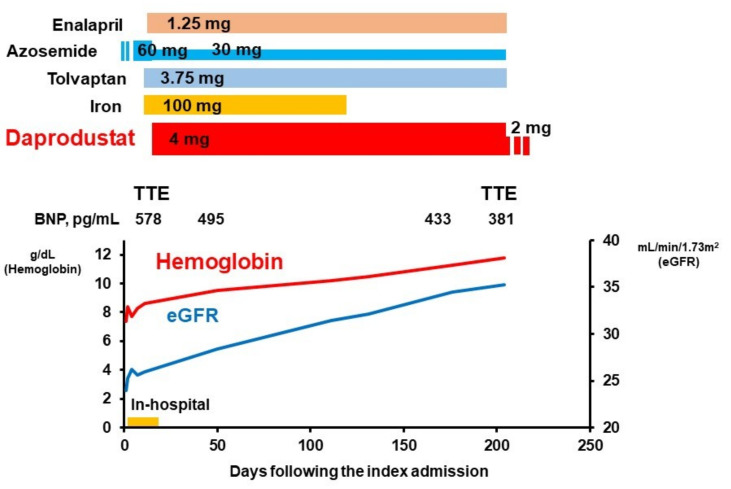
Eight-month time course following the index admission. TTE, transthoracic echocardiography; BNP, B-type natriuretic peptide; eGFR, estimated glomerular filtration ratio.

We initiated enalapril 1.25 mg/day. Tolvaptan 3.75 mg/day was initiated and azosemide was decreased to 30 mg/day (Figure 2). Following the stabilization of congestion, right heart catheterization and coronary angiography were performed. There was no significant coronary artery stenosis. Pulmonary artery wedge pressure was 8 mmHg, mean right atrial pressure was 2 mmHg, and cardiac index was 2.09 L/min/m^2^ before the index discharge.

Fecal occult blood test was negative. He further received gastrointestinal endoscopy, which found grade-A gastroesophageal reflux disease without no active bleeding. Proton pump inhibitor esomeprazole 20 mg/day was initiated.

We diagnosed iron deficiency anemia (without current active bleeding) and renal anemia as dominant causes of his low hemoglobin level. We initiated iron supplement 100 mg/day and daprodustat 4 mg/day.

Just before the index discharge, six-minute walk distance was 324 m and the barthel index was 80 points.

### 2.3. Post-Discharge Course

We followed up him for seven months without any comorbidities including thromboembolic events. Daprodustat 4 mg/day was continued for 7 months. Iron supplement was terminated at 4 months given ferritin 76 ng/mL and transferrin saturation was 28.6%. Hemoglobin level increase gradually up to 11.8 g/dL and daprodustat was decreased to 2 mg/day at 7 months. Estimated glomerular filtration ratio increased up to 35 mL/min/1.73 m^2^ at 7 months without proteinuria. Transthoracic echocardiography at 7 months showed left ventricular end-diastolic diameter 52 mm and left ventricular ejection fraction 54%. The grade of mitral and tricuspid regurgitation improved to mild. The diameter of inferior vena cava was 22/11 with significant respiratory change. New York Heart Association functional class improved to II. Blood pressure trended around 120/70 mmHg. Six-minute walk distance was 364 m. The barthel index was 90 points.

## 3. Discussion

### 3.1. Cause of Anemia

Patients with heart failure often have multiple causes of anemia [6]. Of them, iron deficiency is a major cause of anemia. Our patient had iron deficiency with a low ferritin saturation (<20%) on admission. We administered oral iron supplement instead of trans-venous infusion given the ameliorated gastrointestinal congestion.

Blood dilution due to systemic congestion might have contributed to the anemia. However, hemoglobin level did not increase considerably despite amelioration of congestion during the index hospitalization. We cannot deny the impact of chronic inflammation due to cardiac cachexia on anemia, known as cardio-anemia syndrome [7].

We considered the concomitant renal anemia as a major cause of anemia in this patient given the impaired renal function and insufficiently elevated erythropoietin level [2].

### 3.2. Impact of HIF-PH Inhibitor on Renal Function

We preferred orally administrable HIF-PH inhibitor to conventional intravenous/subcutaneous administration of erythropoietin-stimulating agents because the patient was not dependent on hemodialysis and followed at the out-patient clinic.

Furthermore, the clinical benefit of erythropoietin-stimulating agent therapy in patients with heart failure remains controversial. Several randomized control trials demonstrated that erythropoietin-stimulating agents did not reduce cardiovascular events or were rather harmful [8,9]. The impact of HIF-PH inhibitors on heart failure patients has not yet been fully investigated thus far.

As observed in our patient, a 7-month daprodustat therapy increased hemoglobin level against the existence of heart failure-related chronic inflammation [7]. Although further studies are warranted, daprodustat might be a promising therapeutic tool to ameliorate renal anemia also in the heart failure cohort.

Furthermore, renal function improved during the 7-months daprodustat therapy. Kuriyama and colleagues previously demonstrated that the administration of recombinant erythropoietin improved renal function in patients with chronic kidney disease [3]. They hypothesized that improvement in renal tissue hypoxia and endothelial cell dysfunction by incremental hemoglobin level might have reno-protective effect.

Improvement in anemia might have contributed to the cardiac reverse remodeling, which increases renal blood flow, suppresses renin-angiotensin-aldosterone system, and ameliorates renal congestion. These might also have indirectly contributed to the reno-protection.

### 3.3. Future Concern

The hemoglobin level was maintained around 11 g/dL, but an ideal hemoglobin target, at which patients can enjoy maximum reverse remodeling and minimum mortality and morbidity, remains the next concern. The impact of HIF-PH inhibitors on other clinical outcomes including exercise capacity remains a future concern. The applicability of our finding using daprodustat on other HIF-PH inhibitors or other renal diseases is uncertain.

This is just a single case report and we cannot derive any definitive conclusions from this case alone. We believe that this is a proof-of-concept and would like clinicians focus on similar cases to accumulative clinical data and conduct large-scale studies, validating our findings and constructing therapeutic strategy using HIF-PH inhibitors for those with heart failure and renal anemia.

## Data Availability

Data are available upon reasonable request.
